# Transient loss of consciousness immediately after total pancreatectomy for pancreatic metastases from renal cell carcinoma: a case report

**DOI:** 10.1186/s40792-022-01583-7

**Published:** 2023-01-09

**Authors:** Yasutaka Masuda, Sho Kiritani, Junichi Arita, Akihiko Ichida, Yoshikuni Kawaguchi, Nobuhisa Akamatsu, Junichi Kaneko, Kiyoshi Hasegawa

**Affiliations:** Hepato-Biliary-Pancreatic Surgery Division, Department of Surgery, Graduate School of Medicine, The University of Tokyo, 7-3-1 Hongo, Bunkyo-Ku, Tokyo, 113-8655 Japan

**Keywords:** Total pancreatectomy, Pancreatic metastasis, Transient loss of consciousness, Mixed acid–base disorder, Postoperative management

## Abstract

**Background:**

Total pancreatectomy (TP) is often selected for treatment of various pancreatic diseases. However, the resultant lack of autoregulation of glycometabolism necessitates careful postoperative management.

**Case presentation:**

A 77-year-old man who had undergone right nephrectomy for renal cell carcinoma 11 years previously presented with multiple histologically diagnosed pancreatic metastases. The patient had no notable comorbidities, including diabetes. Because no extrapancreatic organ metastasis was identified, he underwent TP as a curative treatment. He awoke from anesthesia and was extubated without any problems in the operating room. However, 15 min after entering the intensive care unit, he suddenly lost consciousness and became apneic, resulting in reintubation. Blood gas analysis revealed an increased glucose concentration (302 mg/dL) and mixed acid–base disorder (pH of 7.21) due to insulin insufficiency and fentanyl administration. After induction of continuous intravenous insulin infusion and termination of fentanyl, the glucose concentration and pH gradually improved. He regained clear consciousness and spontaneous ventilation and was extubated the next day with no difficulties or complications.

**Conclusion:**

This case highlights the importance of active monitoring of the glycemic state and pH after TP because of the possibility of deterioration due to TP itself as well as the lingering effects of anesthesia.

## Introduction

Total pancreatectomy (TP) is a viable option that is mainly applicable to malignant pathologies of the pancreas [1]. This procedure was first reported by Rockey in 1943 [2]. It was initially abandoned because of high perioperative morbidity and mortality rates, but it has since become a widely selected option because of improvements in both surgical techniques and postoperative management [1–3].

The primary hormones of the pancreas include insulin and glucagon, and both are responsible for the homeostasis of glycometabolism [4–6]. TP results in drastic glycemic instability with a requirement for insulin therapy [1, 7]. Diabetic ketoacidosis and hypoglycemic episodes are well-known complications related to glycemic instability, and their management has been studied [8]. Regarding the short-term complications after TP, morbidity rate has been reported to vary 25 to 60% [9–11]. Transient loss of consciousness (TLOC) sometimes occurs mainly derived from postoperative hypoglycemic state, not hyperglycemic state [8–10]. We herein describe a patient who developed TLOC accompanied by hyperglycemic state immediately after TP.

## Case presentation

**Fig. 1:**
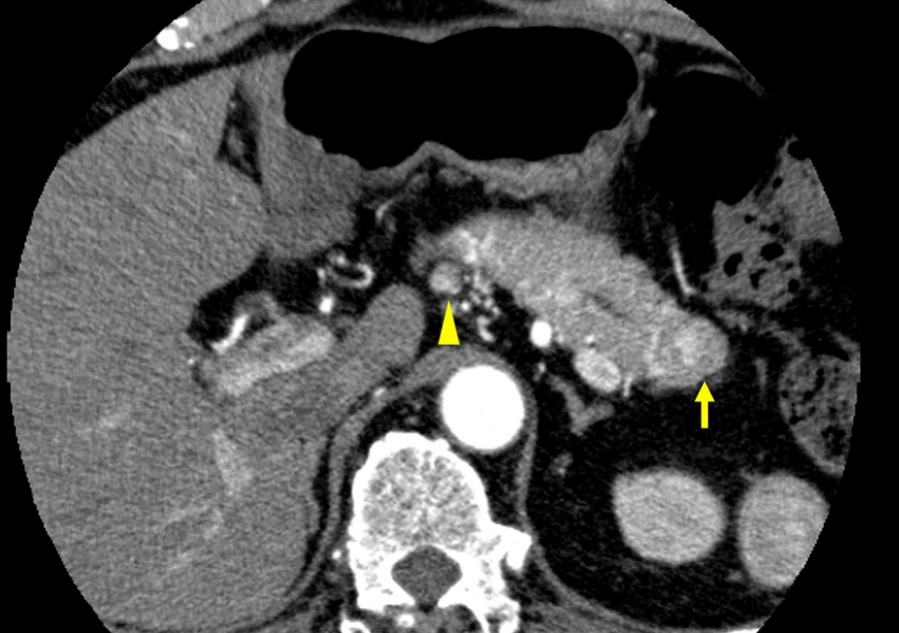
Contrast-enhanced computed tomography images before the operation. Hypervascular lesions were identified in the pancreatic parenchyma (yellow arrow), and a regional lymph node was swollen (yellow arrowhead)

A 77-year-old man presented with multiple pancreatic tumor lesions with a maximum diameter of 13.8 mm. The lesions had been identified by periodic ultrasonographic check-ups. He had undergone laparoscopy-assisted right nephrectomy for renal cell carcinoma 11 years previously and had since been free of tumor recurrence. His medical history also included dyslipidemia; however, he had no history of diabetes. Laboratory data showed no remarkable changes, including changes in tumor markers. Contrast-enhanced computed tomography (CT) revealed multiple (at least 10) hypovascular tumors located in the whole pancreas and swollen regional lymph nodes (Fig. 1). Endoscopic ultrasonography depicted these lesions as hypoechoic tumors, and fine-needle aspiration proved that they were pancreatic metastases from renal cell carcinoma. Extrapancreatic organ metastasis was not detected by positron emission tomography. Thus, TP with regional lymph node dissection was performed as a curative treatment. The pancreas was soft. The operation time was 425 min, and the blood loss was 510 mL.

**Fig. 2:**
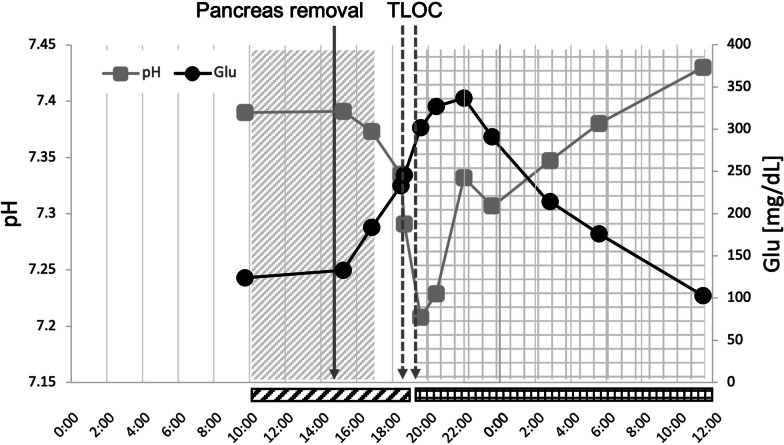
The patient’s clinical course. The diagonally shaded area represents the operation time. The grid area represents the cannulation time. The diagonally shaded and grid rectangular areas below the x axis represent the intravenous fentanyl and insulin administration time, respectively. *Glu* glucose, *TLOC* transient loss of consciousness

**Fig. 3:**
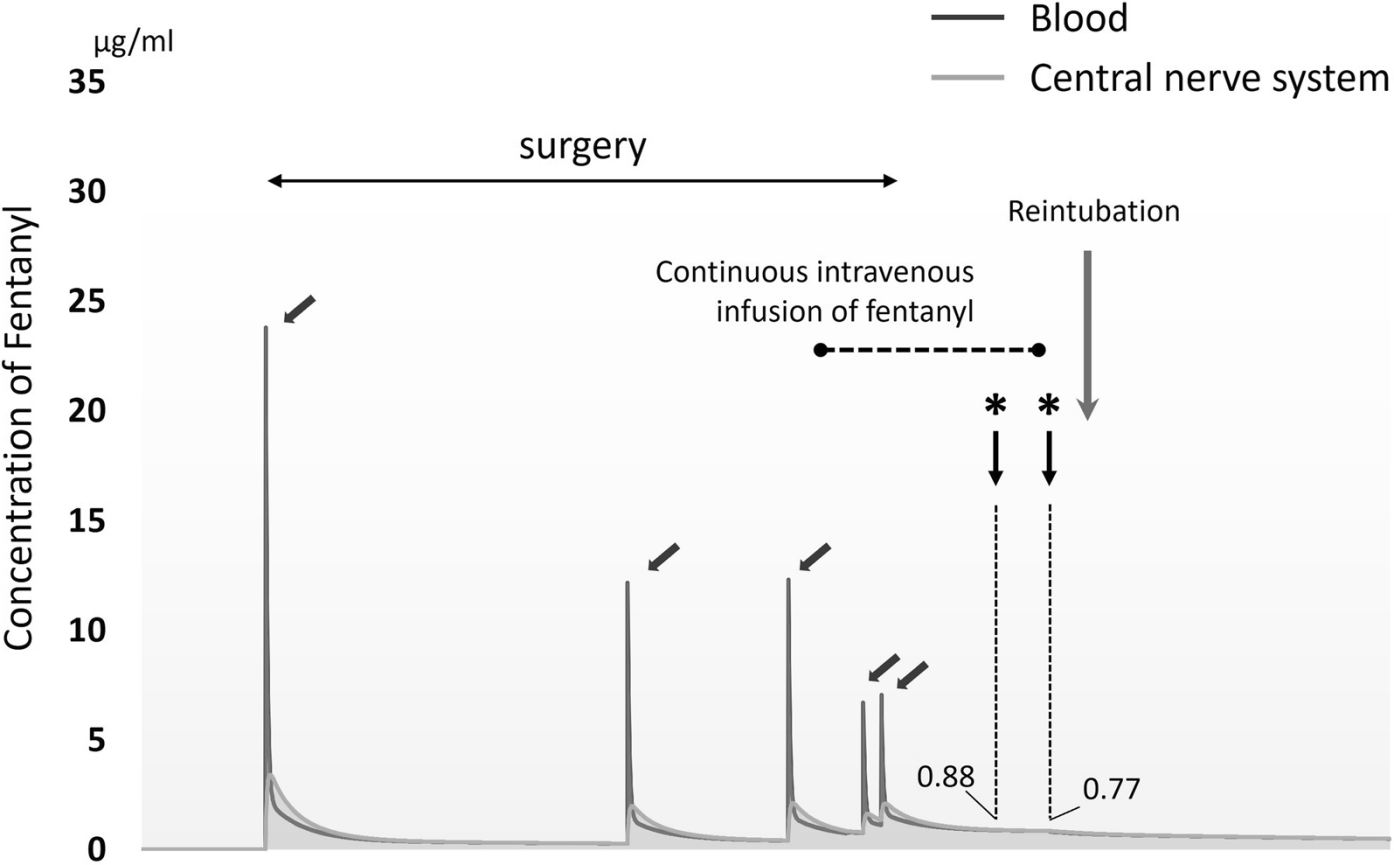
Estimated concentration of fentanyl at the central nerve system (grey curve) and plasma (black curve) drawn by Tivatrainer software. The logo “*” shows the timing of TLOC. Diagonal arrow shows the shot of fentanyl. The fentanyl concentration of central nerve system at the TLOC was 0.88 and 0.77 ng/ml, respectively. *TLOC* transient loss of consciousness

Arterial blood gas analysis at the end of the operation was within normal limits (pH, 7.37; pCO_2_, 36.0 mmHg; HCO_3_, 20.5 mEq/L; and base excess, − 3.9). After the patient had recovered from general anesthesia, he was transferred from the operating room to the intensive care unit. His vital signs and consciousness were normal immediately after this transfer. Fifteen minutes after entering the intensive care unit, however, he suddenly and completely lost consciousness and stopped breathing with no prodrome. His oxygen saturation dropped to 34% despite oxygenation at 3 L/min using a face-covering mask; therefore, high-flow oxygen was administered. He spontaneously regained consciousness after 4 min. Arterial blood gas analysis during this episode revealed mild metabolic acidosis (pH, 7.34; pCO_2_, 42.6 mmHg; and HCO_3_, 22.1 mEq/L). His lactate (2.6 mmol/L) and glucose (233 mg/dL) concentrations were higher than upon completion of the operation. Intravenous administration of fentanyl was terminated because of the concern that it may prevent spontaneous ventilation. Ten minutes after recovery of consciousness, the acidosis became exacerbated (pH, 7.29; pCO_2_, 44.5 mmHg; and HCO_3_, 20.8 mmHg). The patient’s lactate (3.1 mmol/L) and glucose (246 mg/dL) concentrations increased further. Twenty minutes after the first episode, he lost consciousness and stopped breathing again. Because his consciousness did not improve (unlike the first episode), reintubation for forced ventilation was performed. Blood gas analysis showed severe acidosis and hyperglycemia (pH, 7.21; pCO_2_, 55.9 mmHg; HCO_3_, 21.4 mmHg; base excess, − 6.7; lactate, 2.9 mmol/L; and glucose, 302 mg/dL). His serum ketone (β-hydroxybutyrate) concentration after the second episode of TLOC was high (0.713 mmol/L). Head CT after reintubation revealed no remarkable change. An electrocardiogram and transthoracic echocardiogram revealed no arrhythmia or abnormal cardiac function. Continuous electrocardiographic monitoring and arterial pressure measurement throughout the episodes showed no arrhythmia or cardiac shock. Continuous insulin infusion was initiated immediately after the second episode of TLOC. The patient’s blood glucose and pH returned to the normal range, and he regained consciousness and spontaneous ventilation. The patient’s clinical course is summarized in Fig. 2. Estimated concentration of fentanyl at the central nerve system and plasma analyzed by Tivatrainer (version 9.1, Digital River GmbH Scheidtweilerstr, Cologne, Germany) is shown in Fig. 3. The next day, he was extubated without needing reintubation. An electroencephalogram recorded on postoperative day (POD) 10 revealed no signs of epilepsy or other neurological diseases. Head magnetic resonance imaging and angiography on POD 11 were devoid of sites of infarction or signs of epilepsy, and the blood vessels in the head and neck were clearly depicted without narrowing. On POD 31, the patient was discharged from the hospital with no severe postoperative complications besides the above-mentioned episodes. The final pathological diagnosis was metastases of clear cell renal cell carcinoma to the pancreas and surrounding lymph nodes with a negative surgical margin. He underwent no adjuvant therapy, and he was alive without recurrence 6 months after surgery. At the time of this writing, he had experienced no loss of consciousness since then.

## Discussion

We have herein described a patient who developed TLOC immediately after TP. The most striking feature of this case was the mixed acid–base disorder with hyperglycemic state after pancreatectomy. Although it tends to be hyperglycemic state after TP, TLOC after TP has been reported as a result of hypoglycemic state [8–10]. Only one case was reported to be TLOC and die from diabetic ketoacidosis, but it occurred 5 months after surgery [8]. As far as we know, there has been no report regarding the case of TLOC immediately after TP accompanied by hyperglycemic state. Because the ∆PaCO_2_ was less than expected from the ∆HCO_3_ during the second episode of TLOC, the acidosis was considered to have been influenced by both metabolic and respiratory disorders [12–14]. Although consciousness disorder with hyperglycemic state is the same clinical condition as diabetic ketoacidosis, the serum ketone (β-hydroxybutyrate) concentration and anion gap are usually higher than in the present case [15]. Hyperventilation is a well-known compensatory mechanism in diabetic ketoacidosis, but it was interrupted by lingering effect of fentanyl in this case (pCO_2_ was 55.9 mmHg at the timing of TLOC). Therefore, although serum ketone level was not elevated very much, pH kept decreasing, which resulted in TLOC for this patient. From above the consideration, we considered that the metabolic acidosis and respiratory acidosis both worked on the occurrence of TLOC. The acidemia improved promptly and spontaneous ventilation recovered after the initiation of continuous insulin infusion and termination of fentanyl, supporting this possibility. Of course, fentanyl is well-known anesthesia medication to cause postoperative loss of consciousness independently. However, the fentanyl concentration of central nerve system analyzed by Tivatrainer software was low and decreasing at the timing of TLOC (Fig. 3). Therefore, fentanyl was not considered to be the only cause of TLOC. In this simulation, the possible change of fentanyl metabolism accompanied by surgery was not incorporated. However, the fentanyl metabolism was not considered to be altered through the TP, because the hepatic blood flow, which was thought to play the greatest effect on fentanyl metabolism [16], was not changed by this procedure. Also, the renal function of this patient was slightly deteriorated because of previous nephrectomy. However, the proportion of renal extraction of fentanyl is less than 8.0% [17], thus, the influence of which on fentanyl metabolism seemed low.

Other possible causes of TLOC include syncope and epileptic seizure [18–20]; however, both were unlikely in our patient. After the operation, continuous monitoring revealed stable vital signs and electrocardiographic indices. Furthermore, preoperative/postoperative CT and magnetic resonance imaging showed no signs of vascular disease, and the patient had no signs of syncope. TLOC due to epileptic seizure was also unlikely because he did not present with convulsions, and his serum lactate concentration remained stable. Electrocardiographic examination and magnetic resonance imaging on POD 10 and 11 showed no remarkable abnormalities.

TP changes glycometabolism in a dynamic manner because of the lack of glucose-raising and -lowering hormones. Despite the high frequency and severity of pancreatogenic diabetes after TP, no generally accepted guidelines on its management have been established [9, 21]. The American Diabetes Association suggests classification of pancreatogenic diabetes as type 3c and proposes glycemic management according to the guideline for type 1 diabetes; i.e., induction of continuous insulin infusion at 0.1 U/kg/h is recommended [9, 22–24]. In the present case, the continuous insulin infusion was not initiated until the second episode of TLOC because the postoperative blood glucose level was maintained at 140 to 190 mg/dL. Previous reports have indicated that intensive glucose control increases mortality among patients in the intensive care unit [25]. By contrast, some authors have proposed a target postoperative glucose level of < 140 mg/dL, which is associated with low rate of postoperative complications [21, 26, 27]. Thus, the optimal range of the glucose level after TP may be narrow, and frequent glucose checks are required. In this case, earlier intravenous insulin infusion with tight glycemic control might have prevented repeated TLOC. In Japan, perioperative safe and strict glycemic control using bedside artificial pancreas system has been developed since 2010 [28]. In this case, earlier insulin administration might prevent TLOC. Thus, more aggressive introduction of an artificial pancreas should be considered.

## Data Availability

All data generated or analyzed during this study are included in this article. Further inquiries can be directed to the corresponding author.

## Abbreviations

TP: Total pancreatectomy
TLOC: Transient loss of consciousness
CT: Computed tomography
POD: Postoperative day
